# Supplementary material for: Global hypo-methylation in a proportion of glioblastoma enriched for an astrocytic signature is associated with increased invasion and altered immune landscape
Source: eLife. 2022 Nov 22;11:e77335. doi: 10.7554/eLife.77335 (PMC9681209; doi:10.7554/eLife.77335)
Supplement: Figure 2—source data 1. [file elife-77335-fig2-data1.zip › Figure_2_source_data_1/Figure_2C/homerResults/motif83.similar.html]

motif83

## Information for motif83

T
C
G
A
T
C
A
G
T
A
G
C
A
T
G
C
G
A
T
C
A
G
C
T
A
G
C
T
G
T
A
C
A
G
T
C
A
T
G
C
G
A
T
C
G
A
T
C
A
T
C
G
C
T
A
G
A
T
C
G
A
C
T
G
A
C
G
T
C
T
A
G
T
C
G
A
T
C
A
G
A
T
G
C
T
A
G
C
G
A
T
C
A
G
C
T
A
G
C
T
G
T
A
C
A
G
T
C
A
T
G
C
G
A
T
C
G
A
T
C
A
T
C
G
C
T
A
G
A
T
C
G
A
C
T
G
A
C
G
T
  
Reverse Opposite:  

T
G
C
A
A
G
T
C
A
T
G
C
G
A
T
C
T
A
G
C
C
T
A
G
C
T
A
G
A
T
C
G
C
T
A
G
C
A
T
G
C
T
G
A
C
T
G
A
C
T
A
G
A
T
C
G
T
A
C
G
A
G
T
C
A
G
C
T
G
A
T
C
G
T
C
A
G
T
A
C
A
T
G
C
G
A
T
C
T
A
G
C
C
T
A
G
C
T
A
G
A
T
C
G
C
T
A
G
C
A
T
G
C
T
G
A
T
C
G
A
C
T
A
G
A
T
C
G
A
T
C
G
A
G
T
C
A
G
C
T
  

|  |  |
| --- | --- |
| p-value: | 1e-2 |
| log p-value: | -6.472e+00 |
| Information Content per bp: | 1.654 |
| Number of Target Sequences with motif | 13.0 |
| Percentage of Target Sequences with motif | 1.11% |
| Number of Background Sequences with motif | 20.9 |
| Percentage of Background Sequences with motif | 0.43% |
| Average Position of motif in Targets | 95.2 +/- 47.4bp |
| Average Position of motif in Background | 113.7 +/- 40.7bp |
| Strand Bias (log2 ratio + to - strand density) | 1.2 |
| Multiplicity (# of sites on avg that occur together) | 1.92 |
| Motif File: | file (matrix) reverse opposite |

### Similar de novo motifs found

|  |  |  |  |  |  |  |  |
| --- | --- | --- | --- | --- | --- | --- | --- |
| Rank | Match Score | Redundant Motif | P-value | log P-value | % of Targets | % of Background | Motif file |
| 1 | 0.718 | G T C A C T G A A C G T A T G C A G C T C G A T A G C T A G T C A T G C A G T C C G A T A G C T A C T G A C T G A C T G C T A G C G A T A T C G G T A C C T G A A G T C A T G C A T G C C G A T A G C T T A G C A G T C A G T C C G A T G T A C A C T G A C T G A C T G C A T G G C A T A T C G G T C A A C T G A G T C T G C A A T G C G C A T A G C T T G A C A T G C | 1e-2 | -6.218199 | 0.26% | 0.00% | motif file (matrix) |
